# Towards microbial fermentation metabolites as markers for health benefits of prebiotics

**DOI:** 10.1017/S0954422415000037

**Published:** 2015-06

**Authors:** Kristin A. Verbeke, Alan R. Boobis, Alessandro Chiodini, Christine A. Edwards, Anne Franck, Michiel Kleerebezem, Arjen Nauta, Jeroen Raes, Eric A. F. van Tol, Kieran M. Tuohy, on behalf of the ILSI Europe Prebiotics Task Force Expert Group ‘Microbial metabolism and fermentation’

**Affiliations:** 1 Translational Research in Gastrointestinal Disorders (TARGID), KU Leuven and Leuven Food Science and Nutrition Research Center (LFoRCe), Leuven, Belgium; 2 Department of Medicine, Imperial College London, London, UK; 3 Formerly ILSI Europe, Box 6, Avenue Emmanuel Mounier 83, BE-1200, Brussels, Belgium; now European Commission, Research Executive Agency (REA) Unit B2, Brussels, Belgium; 4 Human Nutrition School of Medicine, College of MVLS, University of Glasgow, Glasgow, Scotland; 5 Cargill, Vilvoorde, Belgium; 6 Host Microbe Interactomics, Wageningen University, Wageningen, The Netherlands; 7 FrieslandCampina, Amersfoort, The Netherlands; 8 Microbiology and Immunology, Rega Institute, KU Leuven, Leuven; VIB, Leuven; DBIT, Vrije Universiteit Brussel, Brussels, Belgium; 9 Mead Johnson Nutrition, Nijmegen, The Netherlands; 10 Nutrition and Nutrigenomics, Research and Innovation Centre-Fondazione Edmund Mach, Trento, Italy

**Keywords:** Microbial metabolites, Prebiotic health benefits, Metagenome, Nutrikinetics

## Abstract

Available evidence on the bioactive, nutritional and putative detrimental properties of gut microbial metabolites has been evaluated to support a more integrated view of how prebiotics might affect host health throughout life. The present literature inventory targeted evidence for the physiological and nutritional effects of metabolites, for example, SCFA, the potential toxicity of other metabolites and attempted to determine normal concentration ranges. Furthermore, the biological relevance of more holistic approaches like faecal water toxicity assays and metabolomics and the limitations of faecal measurements were addressed. Existing literature indicates that protein fermentation metabolites (phenol, *p*-cresol, indole, ammonia), typically considered as potentially harmful, occur at concentration ranges in the colon such that no toxic effects are expected either locally or following systemic absorption. The endproducts of saccharolytic fermentation, SCFA, may have effects on colonic health, host physiology, immunity, lipid and protein metabolism and appetite control. However, measuring SCFA concentrations in faeces is insufficient to assess the dynamic processes of their nutrikinetics. Existing literature on the usefulness of faecal water toxicity measures as indicators of cancer risk seems limited. In conclusion, at present there is insufficient evidence to use changes in faecal bacterial metabolite concentrations as markers of prebiotic effectiveness. Integration of results from metabolomics and metagenomics holds promise for understanding the health implications of prebiotic microbiome modulation but adequate tools for data integration and interpretation are currently lacking. Similarly, studies measuring metabolite fluxes in different body compartments to provide a more accurate picture of their nutrikinetics are needed.

## Introduction

For a long time, the colon was considered as an organ that merely absorbs water and electrolytes and converts undigested food residues to drive their excretion without having important physiological functions. Nowadays, it has been generally recognised that the microbial ecosystem inhabiting the gut profoundly affects human physiology and health. The gut bacteria can be considered as a highly active metabolic organ that provides metabolic traits that complement those encoded within our own genome. For instance, degradation of several structural polysaccharides in plant cell walls requires enzymes that are not encoded by the host but are available in specific bacteria^(^ ^1^ ^)^. The collective genetic information encoded in the intestinal micro-organisms is truly impressive and has been referred to as ‘our other genome’^(^ ^2^ ^)^.

The metabolites produced by the gut bacteria are accessible to the host's cells and in this way influence physiological processes both locally in the intestine and systemically. They contribute to the metabolic phenotype of the host and hence may influence the risk of disease^(^ ^3^ ^)^. Undigested carbohydrate and protein constitute the major substrates at the disposal of the microbiota for fermentation and result in the production of a range of well-established metabolites including SCFA, branched-chain fatty acids (BCFA), ammonia, amines, phenolic compounds and gases including hydrogen, methane and hydrogen sulfide. Other metabolic activities include the activation or inactivation of bioactive food components like isoflavanoids, flavanoids and plant lignans, the conversion of pro-drugs to drugs, the production of vitamins and the transformation of bile acids and xenobiotics^(^ ^4^ ^,^ ^5^ ^)^. Although the fermentative and metabolic activity in the human intestine has been studied for many decades, it remains difficult to evaluate bacterial metabolism in the colon *in vivo*. Most studies have relied on analysis of the composition of faeces, *in vitro* incubation studies using faecal inocula or experimental animal models.

An imbalance in the composition of the microbiota has been increasingly associated with the occurrence of chronic or lifestyle-related diseases such as inflammatory bowel disease (IBD), obesity and type 2 diabetes as well as with certain autoimmune diseases such as type 1 diabetes, coeliac disease or allergic asthma^(^ ^6^ ^)^. Therefore, manipulation of the microbiota has become a promising target for the improvement of host health. As diet is a major factor driving the composition and metabolism of the colonic microbiota, dietary interventions that modulate the supply of macronutrients (carbohydrates, proteins, fat) to the colon have been extensively investigated for this purpose. In particular prebiotics, defined as ‘selectively fermented food ingredients that allow specific changes in composition and/or activity of the microbiota that confer benefits upon host well-being and health’^(^ ^7^ ^)^, have been used in an attempt to improve gut health and by extension systemic health (http://www.worldgastroenterology.org/probiotics-prebiotics). Previously, the effect of prebiotic supplementation has been measured using the relative increase in *Bifidobacterium* and *Lactobacillus* species as markers^(^ ^8^ ^)^. However, increasing knowledge on the intestinal microbiota has shown that other genera or species may also confer health benefits, expanding the potential role of prebiotics. Emerging genera that may play a role in the maintenance of intestinal homeostasis and health include *Eubacterium*, *Faecalibacterium*, *Roseburia* and some species of *Clostridia* ^(^ ^7^ ^)^. Since the microbiota is characterised by a significant degree of functional redundancy^(^ ^9^ ^)^, meaning that different bacteria are able to perform similar functions, metabolise the same substrates and produce similar metabolites, analysing the activity of the microbiota rather than its composition and structure may be more relevant to assess the impact of prebiotic interventions.

In the present review, we have re-evaluated our current understanding of the role of bacterial metabolites in promoting or reducing health to estimate the potential applicability of those metabolites as markers of improved/reduced gut health. Moreover, we recognise that the contribution of the microbiota to the overall mammalian biochemistry may play distinctive roles at different developmental stages of the host. We have identified the limitations associated with analysis of single metabolites and have evaluated the usefulness of more holistic approaches including functional analysis of faecal water, metabolomics and metagenome analysis. The ultimate goal is to move towards a definition of ‘healthy metabolic signatures’ that might comprise integrated measures of metabolite patterns in different matrices.

## Metabolites produced by microbial fermentation

The complex microbial ecosystem inhabiting the human intestinal tract produces a wide range of metabolites that interact with the host's cells and in this way influence the physiological processes in the colon. In addition, the metabolites may be absorbed and influence the overall mammalian biochemistry, thereby eliciting systemic effects. Table 1 provides an overview of the major bacterial compounds that can be found in the intestine.

**Table 1:** List of bacterial metabolites that may be found in the intestine

Type of metabolite	Metabolites
Metabolites derived from bacterial energy metabolism	‘Terminal’ metabolites from carbohydrate fermentationSCFA: formate, acetate, propionate, butyrate, longer-chain fatty acidsBranched-chain fatty acids
	‘Intermediate’ metabolites from carbohydrate fermentationPartially degraded oligomeric carbohydrates (disaccharides, oligosaccharides, complex proteoglycans from mucins, etc.)Alcohols: methanol, ethanol, etc.
	Gaseous metabolitesFermentation gases: hydrogen, methane, carbon dioxideHighly volatile compounds: hydrogen sulfide
	Metabolites of fatty acid and lipid bioconversionLong-chain aldehydesFatty acids
	Metabolites from protein fermentationBranched-chain fatty acidsAmmonia and aminesAromatic derivatives of amino acids: phenols, cresols, indoles, etc.
Metabolites derived from bioconversion of plant secondary compounds	Products of lignin/polyphenols bioconversion: equol, enterolactone, etc.
Metabolites from bacterial cytosolic compartment or secondary metabolism (spilled over by excess production, efflux or upon cell lysis)	Vitamins and cofactors (often in very small concentrations) Peptides (quorum-sensing signals of Gram-positive bacteria) Homoserine lactone (quorum-sensing signals of Gram-negative bacteria) Nucleic acids (free DNA, microRNA, etc.) Bacteriocins
Metabolites of the enterohepatic circulation	Bile acids Cholesterol, coprostanol Hormones and derivatives Glucuronide conjugates
Enzymes	Reductases Glucuronidases Glycohydrolases
Bacterial cell wall components |(of which several are immunoactive)	Lipopolysaccharide Polysaccharide A Peptidoglycan-derived structures Capsular polysaccharides (glycocalix)

From this list of compounds, we selected a subset of metabolites that were considered relevant to improved or decreased health. Most of those metabolites are so-called primary metabolites which comprise products of metabolism that are essential for growth or that are the by-products of energy-yielding metabolism. Secondary metabolites (products which do not have an obvious role in cell metabolism such as vitamins) were not included for further analysis. The metabolites reviewed here include products of carbohydrate fermentation (acetic, propionic and butyric acid as well as lactic acid and succinic acid) and products of protein metabolism (ammonia, BCFA, phenol, amines, *p*-cresol, indole and hydrogen sulfide). In addition, metabolites of plant polyphenols have been included because of their putative health benefits and their bidirectional interaction with the intestinal microbiota.

## Beneficial and harmful effects of relevant metabolites

### Products of carbohydrate fermentation

#### SCFA

SCFA are mainly produced in the colon by bacterial fermentation of carbohydrates that escaped digestion in the small intestine. They are saturated aliphatic organic acids consisting of one to six carbons of which acetate (C2), propionate (C3) and butyrate (C4) are the most abundant ( ≥ 95 %)^(^ ^10^ ^,^ ^11^ ^)^. SCFA production mainly occurs in the proximal part of the colon where the availability of substrates is most abundant. The majority of SCFA (up to 95 %) are rapidly absorbed by the colonocytes resulting in decreasing concentrations from the proximal to distal colon. Only a minor fraction of SCFA (about 5 %) is excreted in faeces^(^ ^12^ ^)^.

Due to the inaccessibility of the human proximal colon for direct investigation and the rapid absorption of SCFA from the colonic lumen, it is extremely difficult to quantify SCFA production rates. Consequently, no systematic evaluation of normal ‘healthy’ production of SCFA is available. Assuming that 50–60 g carbohydrates reach the colon per d, the production of SCFA was estimated at 400–600 mmol/d^(^ ^10^ ^)^. Most studies measure faecal SCFA which are the resultant of their production and absorption. Therefore, faecal SCFA rather indicate losses and do not adequately reflect *in situ* production rates. Table 2 provides an overview of reported values in the literature for total and individual faecal SCFA in adults. Faecal excretion of total SCFA ranges from 60 to 90 μmol/g and might be slightly higher in obese subjects (80–100 μmol/g). SCFA are also detectable in urine, but are the remnant of gut, liver and systemic metabolism and do not reflect colonic generation either. In addition, acetate not only originates from the gut but also from endogenous metabolism, in particular fatty acid oxidation and glucose and/or amino acid metabolism^(^ ^13^ ^,^ ^14^ ^)^. Measurement of SCFA in plasma is similarly confounded. Stable isotope studies are required to reliably quantify colonic SCFA production as well as their metabolic fate in the host organism.

**Table 2:** Faecal concentration of individual SCFA

	Subjects (*n*); age	Reported measure	Acetic acid	Propionic acid	Butyric acid	Total SCFA	Unit	Reference
Healthy subjects	10; 21–34 years	Mean (sd)	218 (99)	72 (37)	58·7 (54·5)	378 (188)	μmol/g dry weight	Whelan (2005)^(^ ^236^ ^)^
	20; 20–40 years	Mean (sem)	320·3 (24·9)	97·3 (10·5)	93·8 (9·13)	511·4 (41·9)	μmol/g dry weight	Boler (2011)^(^ ^111^ ^)^
	13; 23–58 years	Median (IQR)	52·2	23·2 (13·6–37·3)	36·8 (5–128)	119·3 (64·5–197·0)	μmol/g wet weight	Lewis (1997)^(^ ^237^ ^)^
	60; 18–24 years	Mean (sem)	198·4 (14·2)	55·2 (4·7)	50·5 (4·9)	304·1	μmol/g dry weight	Lecerf (2012)^(^ ^109^ ^)^
	27; 18–55 years	Mean (sem)	35·8 (2·4)	11·4 (1·2)	10·0 (1·1)	61·1 (4·4)	μmol/g	Reimer (2012)^(^ ^238^ ^)^
	12; 18–65 years		48	13·98	13·31	80·91	μmol/g	Fernando (2010)^(^ ^239^ ^)^
	46; 31–66 years	Mean (95 % CI)	44·7 (39·7, 50·3) females 58·6 (49·8, 69·0) males	12·3 (10·7, 14·0) females 16·1 (13·4, 19·5) males	11·7 (9·8, 14·0) females 15·4 (12·1, 19·6) males	69·5 (61·3, 78·7) females 90·5 (76·3, 108) males	μmol/g	McOrist (2011)^(^ ^240^ ^)^
	36	Median (IQR)	43·7 (34·0–52·2)	13·1 (9·2–18·5)	8·8 (5·2–11·5)	91·8 (73·1–107·5)	μmol/g	Nemoto (2012)^(^ ^241^ ^)^
	20; 22–55 years	Mean (sem)	42·13 (3·84)	11·5 (1·19)	11·28 (1·42)	67·3 (6·22)	μmol/g	Tiihonen (2010)^(^ ^67^ ^)^
	8; 31–59 years	Mean (sd)	NR	NR	NR	92·7 (33·9)	μmol/g	McOrist (2008)^(^ ^242^ ^)^
	20; 23–28 years	Mean (sem)	NR	NR	NR	78·5 (6·4)	μmol/g	Hylla (1998)^(^ ^243^ ^)^
	30	Mean (sd)	50·5 (12·6)	13·6 (5·2)	14·1 (7·6)	84·6 (22·9)	mmol/l	Schwiertz (2010)^(^ ^64^ ^)^
Obese subjects	20; 22–55 years	Mean (sem)	47·15 (3·80)	13·64 (1·34)	14·73 (1·47)	78·79 (6·19)	μmol/g	Tiihonen (2010)^(^ ^67^ ^)^
	91	Mean (sd)	58·5 (19·1)	17·6 (7·6)	18·3 (9·7)	102 (33·5)	mmol/l	Brinkworth (2009)^(^ ^63^ ^)^
	32; 20–65 years	Mean (sem)	NR	NR	NR	34 (6)	mmol/24 h	Benassi Evans (2010)^(^ ^244^ ^)^
	35 overweight 33 obese	Mean (sd)	56·0 (18·2) 59·8 (18·3)	18·3 (7·9) 19·3 (8·7)	18·5 (10·1) 18·1 (10·0)	98·7 (33·9) 103·9 (34·3)	mmol/l	Schwiertz (2010)^(^ ^64^ ^)^

IQR, interquartile range; NR, not reported.

**Fig. 1:**
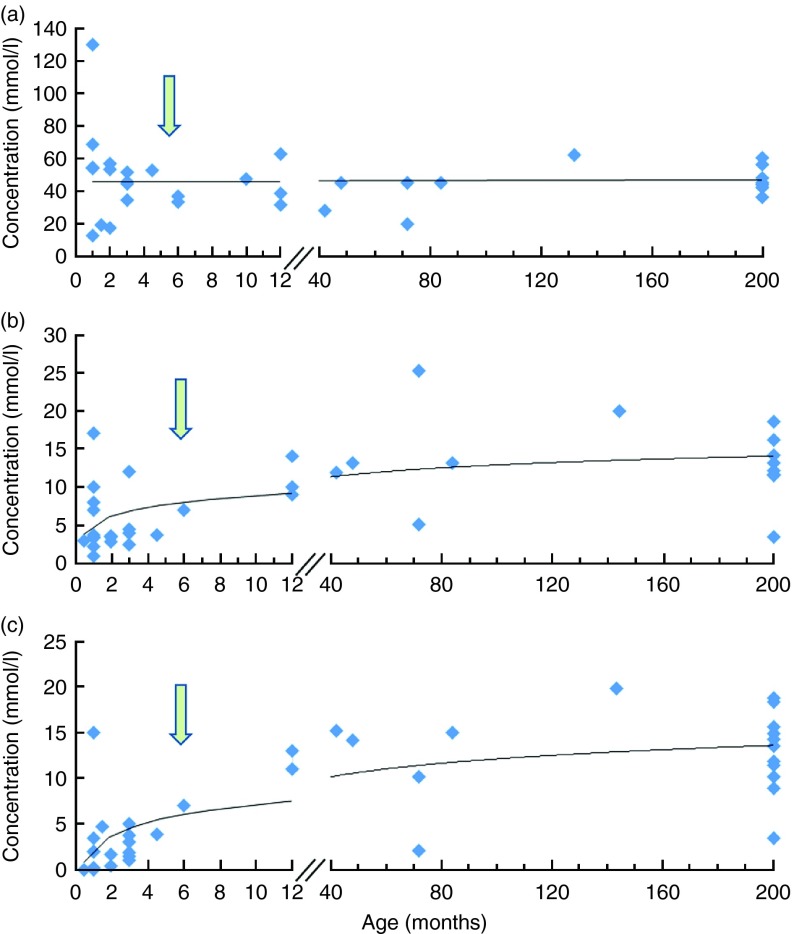
Evolution of faecal SCFA as a function of age: acetic acid (a); propionic acid (b); butyric acid (c). The arrows roughly indicate the change from breast-feeding to solid food with concurrent successional development of the gut microbiota away from one dominated by the bifidobacteria, which produce acetate and lactate during carbohydrate fermentation, to a more complex microbiota with higher relative abundance of Firmicutes, which produce acetate, propionate and butyrate as major SCFA endproducts of carbohydrate fermentation. The figures summarises the data reported in several studies^(^ ^15^ ^,^ ^17^ ^,^ ^71^ ^,^ ^84^ ^,^ ^126^ ^,^ ^228^ ^–^ ^235^ ^)^. A colour version of this figure can be found online at http://www.journals.cambridge.org/nrr

The pattern and amounts of faecal SCFA change through the different stages in life. In early infancy, the predominant SCFA are acetate and lactate in breast-fed infants and acetate and propionate in (unsupplemented) formula-fed infants^(^ ^15^ ^)^. In infants fed a formula supplemented with a mixture of galacto-oligosaccharides and fructo-oligosaccharides (9:1 ratio), faecal SCFA patterns were dominated by acetate, similarly as in breast-fed infants, with lower proportions of propionate and butyrate compared with the unsupplemented formula^(^ ^16^ ^)^. The levels of propionate have been reported to increase in the months before weaning. Butyrate production increases in the second part of the first year of life when faecal lactate levels fall to negligible values (CA Edwards, unpublished results). By the age of 2 years the pattern becomes more similar to that observed in adults^(^ ^17^ ^)^. Fig. 1 depicts the changes in SCFA from birth up to adulthood. In the elderly, the microbiota changes, with higher levels of Bacteroidetes^(^ ^18^ ^)^, which is likely to affect SCFA production. Nevertheless, no differences were detected in SCFA levels in a group of French 68- to 89-year-olds compared with a group of 30- to 46-year-olds^(^ ^19^ ^)^. In contrast, among participants in the pan-European project on the elderly gut microbiota (CROWNALIFE), elderly Europeans (76 ± 7·5 years; *n* 55) had lower concentrations of propionate, acetate and butyrate (by 30, 35 and 21 %, respectively) compared with younger adults (40 ± 9·7 years; *n* 53)^(^ ^20^ ^)^. With these apparently contradicting results obtained in different studies, it remains to be established what the normal patterns of SCFA in faecal material are during different stages of life.

After uptake in the colonocytes, a considerable part of the SCFA is used as an energy source and is oxidised to carbon dioxide and ketone bodies^(^ ^21^ ^)^. The fraction that is not consumed by the colonocytes is transported across the basolateral membrane and reaches the liver via the portal bloodstream. Acetate is used by the liver as a precursor for the synthesis of cholesterol and long-chain fatty acids^(^ ^22^ ^)^. However, in individuals following a Western-type diet, high in refined carbohydrates, sugars and fatty acids and low in fibre, colonic acetate is likely to contribute only little to hepatic lipogenesis. A recent study in obese individuals even showed an inverse association between serum acetate levels and visceral adipose tissue^(^ ^23^ ^)^. In mice, it was shown that acetate derived from colonic fermentation of fermentable carbohydrates crosses the blood–brain barrier and directly suppresses appetite through central hypothalamic mechanisms^(^ ^24^ ^)^. Propionic acid is often the second most predominant SCFA and has received much attention for its potential roles in the reduction of lipogenesis, cholesterol synthesis inhibition, and more recently for its activation of G protein-coupled receptors (GPR) 41 and GPR43, release of satiety hormones and other metabolic and anti-inflammatory effects^(^ ^25^ ^–^ ^27^ ^)^. Butyric acid has been studied for its ability to promote colonic healing in colitis^(^ ^28^ ^)^ and its potential anti-cancer effects^(^ ^29^ ^)^, including apoptosis stimulation^(^ ^30^ ^)^, in part by inhibiting histone de-acetylase^(^ ^31^ ^)^. It has also been shown to inhibit oxidative damage in cultured cancer cells^(^ ^32^ ^)^ and it may improve gut barrier function^(^ ^33^ ^)^. Recent studies in mice have shown that oral administration of propionate and butyrate, but not acetate, facilitates the extra-thymic *de novo* generation of anti-inflammatory regulatory T (T_reg_) cells. In contrast, rectal administration of acetate and propionate, but not butyrate, promoted accumulation of T_reg_ cells, suggesting that butyrate promotes *de novo* generation but not colonic accumulation of T_reg_ cells, whereas acetate has an opposite activity and propionate is capable of both^(^ ^34^ ^)^. Furthermore, propionate and butyrate were shown to activate intestinal gluconeogenesis (IGN), which has beneficial effects on glucose and energy homeostasis, via complementary mechanisms. Whereas butyrate directly activates the IGN genes, propionate-mediated induction of IGN depends on a gut–brain communication axis involving the fatty acid receptor GPR41^(^ ^35^ ^)^. Those beneficial activities of SCFA produced in the intestine have been shown in different animal species including laboratory animals and production/farm animals^(^ ^36^ ^–^ ^38^ ^)^. However, in human subjects the relevant body of evidence is limited mainly because SCFA are traditionally only measured in faeces or fasting blood samples.

*In vitro* fermentation studies with prebiotics or dietary fibre have consistently resulted in increased levels of SCFA. In contrast, several human prebiotic intervention studies failed to demonstrate increased faecal SCFA, most likely due to the rapid colonic absorption of the SCFA, preventing them from being excreted in faeces^(^ ^39^ ^–^ ^44^ ^)^. The relative proportions of the SCFA vary between individuals and are particularly sensitive to the type of carbohydrate being fermented^(^ ^45^ ^,^ ^46^ ^)^. For example, the proportion of propionic acid production is increased during fermentation of guar gum, long-chain arabinoxylans, oats and oat fractions (oat bran and β-glucan), pectin, pulses, wheat dextrin and pyrodextrins^(^ ^47^ ^–^ ^56^ ^)^ whereas oligofructose predominantly yields acetate^(^ ^57^ ^)^. In contrast, the proportion of butyrate production increases with fermentation of starch and inulin-type fructans and often results from secondary fermentation of lactate and acetate, so-called cross-feeding between bacteria^(^ ^58^ ^)^. Indeed, this type of microbial cross-feeding could be viewed as an important physiological function supporting microbiota homeostasis and species richness, which have both been associated with gut health. In view of the different effects of acetic, propionic and butyric acids, the relative proportions of the acids produced are probably as relevant as their total levels.

Several animal and human studies have recently demonstrated an association between the gut microbiota composition and obesity. Initial studies found with a higher Firmicutes:Bacteroidetes ratio in the obese^(^ ^59^ ^,^ ^60^ ^)^. A greater fermentation capacity in both obese animals and human subjects compared with normal-weight subjects was suggested^(^ ^61^ ^–^ ^63^ ^)^ as well as increased concentrations of caecal or faecal SCFA. It was therefore hypothesised that the efficiency of the energy harvest from food was increased in obesity. However, later studies demonstrated that in human subjects, the relationship between the Firmicutes:Bacteroidetes ratio and obesity is less clear and human obesity may be associated with more subtle changes in the microbiota composition^(^ ^64^ ^,^ ^65^ ^)^. In a recent metagenomic study the typical ecological entity of microbiota ‘richness’ was highlighted as a strong determinant in body-weight control rather than its composition *per se* ^(^ ^66^ ^)^. Reported levels of faecal SCFA in obese subjects were higher than in normal-weight subjects (Table 2), although this has not been confirmed in all studies to date^(^ ^67^ ^,^ ^68^ ^)^. In addition, increased faecal SCFA levels do not necessarily indicate higher absorption rates and increased energy harvest by the host. Indeed, uptake of SCFA in colonocytes via the monocarboxylate transporter 1 (MCT-1) receptor is induced by fibre feeding (pectin) and butyrate, and moreover, inhibited by bile acids. Therefore, SCFA absorption might be reduced in the obese^(^ ^69^ ^,^ ^70^ ^)^. Fermentation capacity can be evaluated as *in vitro* production of SCFA from carbohydrates using faeces from healthy individuals and patients^(^ ^71^ ^)^. In pH-controlled faecal batch cultures, similar levels of SCFA were produced from α-gluco-oligosaccharides by microbiota from obese and lean subjects^(^ ^72^ ^)^. Overall, the relationships between the microbiota composition, intestinal SCFA levels and obesity are far from being elucidated. In a nice series of experiments in animals and human subjects, Cani and colleagues demonstrated how modulation of the microbiota by prebiotics controls endogenous glucagon-like peptide 2 production and the endocannabinoid system and contributes to the improvement in gut barrier function during obesity^(^ ^73^ ^–^ ^75^ ^)^. The direct involvement of specific gut bacteria and/or metabolites needs to be further investigated.

In recent studies, the intestinal bacteria have been implicated in the development of IBD and autoimmune diseases such as type 1 diabetes and coeliac disease. These conditions have been consistently characterised by a low abundance of butyrate-producing bacteria^(^ ^76^ ^–^ ^80^ ^)^. Functional analysis of the microbiota revealed remarkably lower levels of faecal SCFA in IBD^(^ ^81^ ^–^ ^83^ ^)^ whereas total SCFA and in particular acetate were found increased in coeliac disease^(^ ^84^ ^–^ ^86^ ^)^. Allergic children had lower faecal levels of propionate and butyrate than non-allergic children^(^ ^87^ ^)^. It remains to be explored to what extent these aberrant SCFA patterns are causative to the disease or can serve as markers of disease.

#### Lactate and succinate

Lactate and succinate are intermediates in the fermentation process of carbohydrates. In healthy conditions, they are further metabolised to acetate or butyrate and propionate, respectively, by cross-feeding species and do not substantially accumulate in the colonic lumen^(^ ^88^ ^)^. Recent evidence suggests that succinate acts as a signal for inflammation^(^ ^89^ ^)^. It stabilises the transcription factor hypoxia-inducible factor-1α (HIF-1α) in activated macrophages. When stabilised, HIF-1α up-regulates several genes including the inflammatory cytokine IL-1β, resulting in exacerbation of inflammation^(^ ^90^ ^)^. In addition, succinate acts as a ligand for GPR91, renamed SUNCR1. In the kidney, succinate-induced activation of GPR91 is reported to regulate the renin–angiotensin system and in dendritic cells, succinate signalling is required for enhanced antigen-presenting function. Increased levels of succinate have been linked to IBD as mice undergoing dextran sulfate sodium-induced colitis were shown to have more succinate in their caecum and faeces^(^ ^91^ ^)^ whereas in colonic tissue from dextran sulfate sodium-induced mice, succinate levels were lower than in control mice^(^ ^92^ ^)^. Therefore, succinate may be an ulcerogenic agent in the gut lumen, leading to mucosal damage and lower succinate levels in colonic tissues.

Lactate has two optical isomers, which are l-lactate and d-lactate. l-Lactate is produced from pyruvate by the enzyme lactate dehydrogenase during normal anaerobic metabolism whereas d-lactate is produced by many commensal bacteria in the colon. Increased levels of d-lactate in plasma and urine have been demonstrated in IBD^(^ ^81^ ^,^ ^93^ ^)^, intestinal ischaemia^(^ ^94^ ^)^, short bowel^(^ ^95^ ^)^ and appendicitis^(^ ^96^ ^)^ and are considered as a marker of dysbiosis and/or increased intestinal permeability. In faecal samples of IBD patients, mainly l-lactate levels are increased^(^ ^80^ ^,^ ^97^ ^,^ ^98^ ^)^, suggesting a mucosal origin of lactate^(^ ^99^ ^)^. As lactate is a potentially important co-substrate for many sulfate-reducing bacteria, increased colonic lactate levels may promote sulfide generation^(^ ^100^ ^)^ which is suspected of inhibiting the β-oxidation of butyrate in the colonocytes (see below).

### Products of protein metabolism

Microbial products from protein metabolism include BCFA, ammonia, phenol, *p*-cresol, indole and hydrogen sulfide^(^ ^101^ ^)^. The toxic potential of these compounds is mainly derived from *in vitro* experiments, in which isolated cells or tissues are directly incubated with individual compounds, or from animal studies. The present review also encompasses the results of oral toxicity tests as a reasonable surrogate for assessing potential systemic effects. Minimally irritating concentrations were assessed as a marker of local effects and exceeded 1 % for all compounds, which is well above the concentrations occurring in the colon. In human studies, there is little evidence for adverse effects of protein fermentation metabolites^(^ ^102^ ^)^. In a recent study in healthy subjects, modulation of the degree of protein fermentation by changing dietary intake did not affect faecal water toxicity^(^ ^103^ ^)^.

An important determinant of the degree of proteolytic *v.* saccharolytic fermentation is the nutrient availability and in particular the ratio of available carbohydrate to nitrogen^(^ ^104^ ^,^ ^105^ ^)^. Therefore, the production of protein degradation products can generally be reduced by increasing the amount of fermentable carbohydrate reaching the colon in the form of resistant starch^(^ ^104^ ^)^ or prebiotic oligosaccharides^(^ ^106^ ^–^ ^112^ ^)^. In contrast, faecal ammonia, phenol and *p*-cresol were not affected after 4 weeks consumption of the polyol isomalt (30 g/d)^(^ ^113^ ^)^.

#### Phenol, p-cresol and indole

The phenolic compounds phenol, *p*-cresol and indole are the major metabolites of bacterial fermentation of the aromatic amino acids tyrosine, phenylalanine and tryptophan. These metabolites are largely and rapidly absorbed by the colonic mucosa cells and are excreted in urine after sulfate or glucuronide conjugation in the mucosa or the liver^(^ ^114^ ^)^. In healthy subjects, these compounds do not accumulate in the body. Therefore, their urinary elimination is often considered as a reliable estimate of their production in the colon^(^ ^105^ ^)^.

Many studies have reported significant interindividual variation in the urinary excretion of *p*-cresol and phenol in healthy adults. Data on ranges in other age groups (children, elderly) are scarce. Reported mean or median values vary between 10 and 55 mg/d for *p*-cresol (Table 3) and between 4 and 7·5 mg/d for phenol (Table 4). In obese individuals, urinary *p*-cresol and phenol levels at baseline were considerably higher than those reported in normal-weight adults (94·9 mg/d and 15·0 g/d for *p*-cresol and phenol, respectively) and decreased upon weight loss^(^ ^63^ ^)^. The levels of urinary *p*-cresol may increase in the very old^(^ ^115^ ^)^.

**Table 3:** Reported excretion of *p*-cresol in urine and faeces

	Biofluid	Subjects (*n*); age	Reported measure	*p*-Cresol excretion	Unit	Reference
Healthy subjects	Urine	11; 35 ± 10 years	Mean (sem)	454 (92)*	μmol/d	Birkett (1996)^(^ ^104^ ^)^
		27; median 25 (IQR 23–29) years	Median (IQR)	168 (93·3–304) and 208 (114–288)	μmol/d	Damen (2012)^(^ ^107^ ^)^
		9; range 19–69 years	Mean (sem)	408·3 (271·3)	μmol/d	Ling (1992)^(^ ^245^ ^)^
		11; 39 ± 11 years	Median (IQR)	532 (250–659)† *p*-cresyl sulfate	μmol × 1·73 m^2^	Patel (2012)^(^ ^246^ ^)^
		32; range 47–95 years	Mean (sd)	510 (358)*	μmol/d	Renwick (1988)^(^ ^118^ ^)^
		10; range 22–45 years 9; range 22–45 years	Mean (sd)	248 (99)* 315 (206)*	μmol/d	De Preter (2004)^(^ ^247^ ^)^
		15; 23 ± 1 years 15; 22 ± 1 years 15; 23 ± 1 years	Mean (sd)	164 (101)* 186 (119)* 187 (96)*	μmol/d	De Preter (2007)^(^ ^248^ ^)^
		10; 21 ± 1 years 9	Median (IQR)	196 (168–322)* 226 (141–368)*	μmol/d	De Preter (2007)^(^ ^249^ ^)^
		20; median 23 (IQR 21–24) years	Median (IQR)	297 (194–437)	μmol/d	Cloetens (2010)^(^ ^108^ ^)^
		12; median 24 (IQR 21–28) years	Median (IQR)	214 (107–315)	μmol/d	Cloetens (2008)^(^ ^250^ ^)^
		20; range 19–41 years	Median (IQR)	297 (239–349)	μmol/d	Windey (2012)^(^ ^103^ ^)^
		19; range 21–53 years	Mean (sem)	218 (58)	μmol/d	Gostner (2006)^(^ ^113^ ^)^
	Faeces	11; range 3–11 years	Mean (sem)	0·54 (0·29)	μmol/g faeces	Adams (1985)^(^ ^119^ ^)^
		11; 35 ± 10 years	Mean (sem)	0·60 (0·07)*	μmol/g faeces	Birkett (1996)^(^ ^104^ ^)^
		112; range 0–1 years	Mean (sd)	0·14 (0·14)	μmol/g faeces	Heavey (2003)^(^ ^251^ ^)^
		15; 23 ± 1 years 15; 22 ± 1 years 15; 23 ± 1 years	Mean (sd)	124 (38)* 161 (53)* 101 (43)*	μmol/72 h	De Preter (2007)^(^ ^248^ ^)^
		16; range 23–66 years	Mean (sem)	58·86 (7·3)	μmol/g faeces	Clarke (2011)^(^ ^252^ ^)^
		20; range 18–24 years	Mean (sem)	0·52 (0·05)*	μmol/g dry weight	Lecerf (2012)^(^ ^109^ ^)^
		19; range 21–53 years	Mean (sem)	0·36 (0·04)*	μmol/g faeces	Gostner (2006)^(^ ^113^ ^)^
		21; range 21–28 years	Mean (sem)	1·5 (0·20)	μmol/g dry weight	Boler (2011)^(^ ^111^ ^)^
Obese	Urine	91, range 24–64 years	Mean (sd)	879 (00) and 524 (259)*	μmol/d	Brinkworth (2009)^(^ ^63^ ^)^
	Faeces	33, range 20–65 years		0·54*	μmol/g faeces	Benassi-Evans (2010)^(^ ^244^ ^)^

IQR, interquartile range.

* Calculated from reported values in mg/d using a molecular mass value for *p*-cresol of 108.

† Calculated from reported values in mg/d using a molecular mass value for *p*-cresyl sulfate of 188.

**Table 4:** Reported excretio of phenol in urine and faeces

	Biofluid	Subjects (*n*); age	Reported measure	Phenol excretion	Unit	Reference
Healthy subjects	Urine	11; 35 ± 10 years	Mean (sem)	53 (0)	μmol/d	Birkett (1996)^(^ ^104^ ^)^
		27; median 25 (IQR 23–29) years	Median (IQR)	47 (37–66) and 43 (35–59)	μmol/d	Damen (2012)^(^ ^107^ ^)^
		9; range 19–69 years	Mean (sem)	73 (45)	μmol/d	Ling (1992)^(^ ^245^ ^)^
		32; range 47–95 years	Mean (sd)	67 (91)	μmol/d	Renwick (1988)^(^ ^118^ ^)^
		15; 23 ± 1 years 15; 22 ± 1 years 15; 23 ± 1 years	Mean (sd)	45 (21) 42 (25) 46 (22)	μmol/d	De Preter (2007)^(^ ^248^ ^)^
		20; median 23 (IQR 21–24) years	Median (IQR)	55 (31–76)	μmol/d	Cloetens (2010)^(^ ^108^ ^)^
		19; range 21–53 years	Mean (sem)	115 (32)	μmol/d	Gostner (2006)^(^ ^113^ ^)^
	Faeces	11; range 3–11 years	Mean (sem)	0·083 (0·034)	μmol/g faeces	Adams (1985)^(^ ^119^ ^)^
		11; 35 ± 10 years	Mean (sem)	0·042 (0·006)	μmol/g faeces	Birkett (1996)^(^ ^104^ ^)^
		112; range 0–1 years	Mean (sd)	0·58 (0·84)	μmol/g faeces	Heavey (2003)^(^ ^251^ ^)^
		15; 23 ± 1 years 15; 22 ± 1 years 15; 23 ± 1 years	Mean (sd)	8·4 (7·1) 21·0 (16·5) 8·8 (6·9)	μmol/72 h	De Preter (2007)^(^ ^248^ ^)^
		16; range 23–66 years	Mean (sem)	0·7 (0·13)	μmol/g faeces	Clarke (2011)^(^ ^252^ ^)^
		20; range 18–24 years	Mean (sem)	0·0003 (0)	μmol/g dry weight	Lecerf (2012)^(^ ^109^ ^)^
		19; range 21–53 years	Mean (sem)	0·058 (0·015)	μmol/g faeces	Gostner (2006)^(^ ^113^ ^)^
Obese	Urine	91, range 24–64 years	Mean (sd)	158 (119) and 159 (184)	μmol/d	Brinkworth (2009)^(^ ^63^ ^)^
	Faeces	33, range 20–65 years		0·037	μmol/g faeces	Benassi-Evans (2010)^(^ ^244^ ^)^

IQR, interquartile range.

* Calculated from reported values in mg/d using a molecular mass value for phenol of 94.

Most studies on the urinary excretion of the indole metabolite indoxyl sulfate, also called indican, report values below 50 mg/d in healthy adults. In patients with liver cirrhosis^(^ ^116^ ^)^ and patients with diabetes^(^ ^117^ ^)^, excretion of indoxyl sulfate is higher (98·2 mg/d in cirrhosis, 65·7 mg/d in diabetics without neuropathy and 114·0 mg/d in diabetics with neuropathy) and correlates with steatorrhoea. In a study in patients with bladder cancer there was no evidence that phenolic microbial metabolites had promoting or co-carcinogenic activity for the human urinary bladder, as the urinary excretion of *p*-cresol, phenol and indoxyl sulfate was not different in the patients as compared with controls^(^ ^118^ ^)^.

Faecal excretion of phenolic compounds is not often reported, but in available studies amounts of 5–8 mg/d for *p*-cresol and 0·25–0·66 mg/d for phenol have been found. Interestingly, faecal excretion of *p*-cresol was 4-fold higher in a group of hyperactive children as compared with control children^(^ ^119^ ^)^.

The effects of phenolic compounds on intestinal cells have been determined mainly in *in vitro* incubation studies. Viability of colonic epithelial cells isolated from human biopsies was decreased after exposure to 1·25 mm-phenol, a physiologically relevant concentration, whereas higher phenol concentrations (20 mm) were required to reduce viability of HT-29 cells^(^ ^120^ ^)^. Notably, cell cultures from ulcerative colitis (UC) patients showed similar sensitivity to phenol exposure as cell cultures from control subjects at all concentrations tested.

Several papers have reported a concentration-dependent increase in paracellular permeability and reduced epithelial barrier function after incubation with phenol (1 μm to 21 mm) of Caco-2-monolayers or SK-CO15 intestinal cells^(^ ^121^ ^,^ ^122^ ^)^. Enhanced permeability was already apparent at concentrations of phenol that did not cause cell death. Similarly, *p*-cresol altered endothelial barrier function in human umbilical vein endothelial cells. In chronic kidney disease patients, *p*-cresol is considered a uraemic toxin. It accumulates in serum and might participate in the endothelial dysfunction that is observed in such patients^(^ ^123^ ^)^.

The European Food Safety Authority recently evaluated the toxicity of phenol following oral administration (http://www.efsa.europa.eu/en/efsajournal/doc/3189.pdf) and established a tolerable daily intake (TDI) of 0·5 mg/kg body weight per d. For a 75 kg individual, the TDI amounts to 37·5 mg/d, which is about 5-fold higher than the amount of phenol generated in the colon (7·5 mg/d) (assuming that urinary excretion rates reflect colonic generation rates). On repeat-dose administration to non-pregnant rats and mice, no consistent effects were seen at doses ≥  250 mg/kg body weight per d.

The Joint FAO/WHO Expert Committee on Food Additives (JECFA) reviewed the oral toxicity of *p*-cresol in 2011. The systemic toxicity of *p*-cresol was evaluated in a 2-year study in rats following dietary administration as a 60:40 mixture of *m*-/*p*-cresol. (http://www.inchem.org/documents/jecfa/jecmono/v64je01.pdf). A no observed adverse effect level of 230 mg/kg body weight per d was identified, based on increased incidence of renal tubule adenomas in male rats at 720 mg/kg body weight per d. Effects seen in other studies (nasal sinuses, forestomach) were attributed to the local irritancy of *p*-cresol and did not reflect its systemic toxicity. Effects observed in a 13-week repeated exposure study in rats by oral administration were probably secondary to the local irritancy of *p*-cresol.

Data on the toxicity of indole are very limited. JECFA reviewed the effects after oral exposure to indole in 2006 (http://www.inchem.org/documents/jecfa/jecmono/v54je01.pdf). Following exposure of rats to indole at a dose of 100 mg/kg body weight per d in the diet for 460 d, signs of moderate reversible anaemia were apparent. No other adverse effects were observed. Rats fed a low-protein diet supplemented with indole (0·25–2 %) showed overall weight loss and growth retardation as well as haemolytic anaemia^(^ ^124^ ^)^.

#### Ammonia

Due to bacterial degradation of unabsorbed and endogenous nitrogenous compounds and endogenous nitrogen recycling, the colonic epithelium is constantly exposed to ammonia in millimolar concentrations^(^ ^125^ ^)^. Faecal ammonia excretion is comparable in overweight adults and normal-weight adults but is clearly lower in infants (Table 5). A recent study reported significantly higher faecal ammonia concentrations in children with autism spectrum disorders (ASD) (42·7 (SE 3·3) μmol/g faeces) compared with control children (32·3 (SE 1·9) μmol/g faeces)^(^ ^126^ ^)^. As elevated plasma ammonia concentrations have also been described in ASD, the authors suggested that higher faecal concentrations might translate into higher plasma ammonia concentrations.

**Table 5:** Reported excretion of ammonia in faeces

Population	Subjects (*n*); age	Reported measure	Ammonia excretion	Unit	Reference
Healthy subjects	11; 35 ± 10 years	Mean (sem)	23·3 (1·5)	μmol/g faeces	Birkett (1996)^(^ ^104^ ^)^
	112; range 0–1 years	Mean (sd)	7·7 (7·1)	μmol/g faeces	Heavey (2003)^(^ ^251^ ^)^
	16; range 23–66 years	Mean (sem)	14·1 (1·7)	μmol/g faeces	Clarke (2011)^(^ ^252^ ^)^
	12; range 27–49 years	Mean (sd)	5·1 (2·5)	μmol/g faeces	Slavin (2011)^(^ ^41^ ^)^
	9; 59 ± 3 years	Mean (sd)	2·9 (0·5)	μmol/g faeces	Bianchi (2010)^(^ ^253^ ^)^
	46; 31–66 years	Mean (95 % CI)	14·6 (12·6, 16·7)	μmol/kg faeces	McOrist (2011)^(^ ^240^ ^)^
	20; range 20–55 years	Mean (sem)	22·6 (1·6)	μmol/g faeces	Tiihonen (2010)^(^ ^67^ ^)^
	8; range 21–60 years	Mean (sd)	35·2 (7·9)*	μmol/g faeces	Shinohara (2010)^(^ ^110^ ^)^
	21; range 21–28 years	Mean (sem)	137·5 (7·82)	μmol/g dry weight	Boler (2011)^(^ ^111^ ^)^
	36; range 22–67 years	Median (IQR)	24·6 (16·5–30·0)	μmol/g faeces	Nemoto (2012)^(^ ^241^ ^)^
Obese	19; range 20–55 years	Mean (sem)	26·6 (2·2)	μmol/g faeces	Tiihonen (2010)^(^ ^67^ ^)^
	121; 24–64 years	Mean (sd)	26·5 (9·2)	μmol/g faeces	Brinkworth (2009)^(^ ^63^ ^)^

IQR, interquartile range.

* Calculated from reported values in mg/d using a molecular mass value for ammonia of 17.

As early as the 1970s, reports on the effects of ammonia on epithelial cells have appeared. Visek (in 1978)^(^ ^127^ ^)^ was the first to report that ammonia alters nucleic acid synthesis, changes the morphology and intermediary metabolism of intestinal cells and reduces the lifespan of cells. After this initial report, several studies evaluated the impact of physiological concentrations of ammonia using isolated colonocytes, cell lines or animal colon tissue (for reviews, see Windey *et al.* ^(^ ^102^ ^)^ and Blachier *et al.* ^(^ ^128^ ^)^).

The European Food Safety Authority reviewed the effects of dietary administration of ammonia (as ammonium chloride) to rats for up to 30 months (http://www.efsa.europa.eu/en/efsajournal/doc/1925.pdf). It was not possible to identity a no observed adverse effect level from any of the studies, which in general used high doses to study the effects of metabolic acidosis, rather than the compound itself. Although a number of effects were observed, these were considered adaptive, and no adverse effects were observed at doses of 1100 mg/kg body weight per d for 30 months.

#### Hydrogen sulfide

Sulfate-reducing bacteria scavenge hydrogen as an electron donor and use sulfate as an oxidising agent for the dissimilation of organic matter. The major endproduct from this reaction is hydrogen sulfide. Luminal concentrations of sulfide are in the range of 1·0–2·4 mmol/l^(^ ^129^ ^)^ whereas faecal concentrations vary from 0·17 to 3·38 mmol/kg faeces^(^ ^130^ ^,^ ^131^ ^)^. It is probable that a large fraction of sulfide is bound to luminal compounds within the intestine.

The toxic potential of hydrogen sulfide on colonic cells has been extensively investigated. Sulfide influences oxidative metabolism of colonic epithelial cells by inhibiting cytochrome oxidase activity which catalyses the reduction of oxygen to water^(^ ^132^ ^,^ ^133^ ^)^. Several lines of evidence also suggest a role of hydrogen sulfide in the aetiology and/or risk of relapse of UC. In experimental animal models, a pathological condition similar to UC can be induced using undigestible sulfates in the form of dextran sulfate sodium or the sulfate-containing carrageenan. Whilst some studies found elevated faecal sulfide levels in patients with UC^(^ ^134^ ^,^ ^135^ ^)^, others did not^(^ ^136^ ^)^. However, detoxification of sulfide by the mucosal thiosulfate sulfurtransferase enzyme to the less toxic thiocyanate is impaired in UC patients^(^ ^137^ ^)^. In addition, a diet characterised by high meat intake as well as a high sulfur or sulfate intake was associated with increased likelihood of relapse in UC patients^(^ ^138^ ^)^. Exposure of non-transformed rat intestinal crypt cells (IEC-18 cells) to sodium hydrogen sulfide (50 μm) caused acute hypoxia and promoted early cell-cycle entry with an associated up-regulation of genes coding for proteins related to proliferative activity^(^ ^139^ ^)^. A series of *in vitro* experiments by Attene-Ramos *et al.* ^(^ ^140^ ^)^ revealed that hydrogen sulfide provokes genomic DNA damage in colonic cancer cells (HT-29 cells) at concentrations of 250 mm. No cellular metabolism was required for sulfide to induce genotoxicity and co-incubation with a radical scavenger reduced DNA damage induced by hydrogen sulfide, suggesting a radical-mediated mechanism^(^ ^141^ ^)^. In non-transformed human intestinal epithelial cells, the expression of genes involved in cell-cycle progression, inflammation and DNA repair response was modulated by sulfide. In particular, expression of the cyclo-oxygenase (COX)-2 gene, which is elevated in most human colorectal cancers (CRC), was significantly up-regulated^(^ ^142^ ^)^. Overexpression of COX-2 may play a decisive role in promoting CRC initiation or progression through the stimulation of angiogenesis, inhibition of apoptosis and increasing the proliferation in intestinal epithelial cells^(^ ^143^ ^)^.

In contrast to those reports on harmful effects, hydrogen sulfide is now also known to be a systemic signalling molecule. It is endogenously produced in micromolar concentrations from cysteine by the action of cystathionine γ-lyase and cystathionine β-synthase. At these low concentrations, it has been proposed that hydrogen sulfide is involved in neuromodulation of chloride secretion, in controlling ileum contractility and in nociception from the large intestine^(^ ^144^ ^)^. Blachier *et al.* proposed as a working hypothesis that any imbalance between levels of free sulfide in the large intestine and the capacity of epithelial cells to metabolise it will result in a loss of normal oxidative cell capacity^(^ ^144^ ^)^.

Information on the effects of oral exposure to hydrogen sulfide is very limited. The US Environmental Protection Agency (EPA) established a reference dose on the basis of effects observed in pigs, but subsequently withdrew this as it was concluded that the effect was irreproducible (http://www.epa.gov/iris/subst/0061.htm). Although the US EPA has established an inhalation reference concentration for hydrogen sulfide, this is based on local effects and hence is not suitable for assessing the systemic toxicity of the compound.

#### Branched-chain fatty acids

The BCFA isobutyrate, 2-methylbutyrate and isovalerate are produced by bacterial fermentation of valine, isoleucine and leucine, respectively. These BCFA constitute approximately 5–10 % of the total SCFA^(^ ^145^ ^)^. Similar to other protein fermentation metabolites, faecal BCFA concentrations are reduced after prebiotic intake^(^ ^106^ ^,^ ^146^ ^,^ ^147^ ^)^. In an *in vitro* incubation experiment with five different epithelial cell lines; minimal concentrations of isovalerate to induce cytotoxicity were lower than the concentrations produced by intestinal bacteria and both isovalerate and isobutyrate were able to induce apoptosis^(^ ^148^ ^)^. Several *in vitro* studies indicate that BCFA affect the exchange of ions in the colon and may act as a regulator of colonic Na^+^ absorption^(^ ^128^ ^)^ but little information is available regarding other effects of BCFA on colonic epithelial cells. Therefore, faecal concentrations of BCFA are considered only as markers for bacterial protein fermentation rather than markers of colonic health^(^ ^149^ ^)^.

### Plant polyphenolic compounds/catabolites

While the physiological relevance of polyphenol catabolites derived from the gut microbiota is currently understudied, it is recognised that the majority of plant bioactive compounds must first be rendered biologically available, often through deglycation and hydrolysis by the gut microbiota before being absorbed by the host, and that microbial metabolic transformation can make an impact on polyphenol biological activity. While certain classes of phytochemical are broken down to unique catabolites, many different classes of polyphenol give rise to common small phenolic compounds. However, we currently do not know the physiological relevance of many of these compounds, or even the habitual or ‘normal’ concentration ranges of these phenolic acids, or how they respond to diet. For a few of these compounds, for example, the phyto-oestrogens equol, enterolactone and enterodiol, and the urolithins, specific health effects have been suggested^(^ ^150^ ^)^. Table 6 provides an overview of the microbial catabolites of common plant polyphenols and their putative health effects. For an up-to-date review on plant polyphenols catabolites and their putative health effects, see Del Rio *et al.* ^(^ ^150^ ^)^ and Dall'Asta *et al.* ^(^ ^151^ ^)^.

**Table 6:** List of microbial catabolites of common plant polyphenols and their putative health effects^(^ ^155^ ^)^

Plant polyphenol	Microbial catabolite	Possible health effects	References
(–)-Epicatechin			4-Hydroxyphenylacetic acid	Antimicrobial/antimycotic activity *in vitro*	Alakomi (2007)^(^ ^254^ ^)^ Ko (2009)^(^ ^255^ ^)^ Roowi (2010)^(^ ^256^ ^)^
3-(3-Hydroxyphenyl)propionic acid	Antimicrobial activity against Gram-negative enterobacteria via outer membrane destabilisation	
5-(3,4-Dihydroxyphenyl)-γ-valeric acid	?	
(-)-5-(3′,4′-Dihydroxyphenyl)-γ-valerolactone	?	
(–)-Epigallocatechin	4-Hydroxyphenylacetic acid	Antimicrobial/antimycotic activity *in vitro*	Roowi (2010)^(^ ^256^ ^)^
(-)-5-(3′,4′-Dihydroxyphenyl)-γ-valerolactone		
(–)-Epigallocatechin-3-*O*-gallate	Pyrocatechol		Ko (2009)^(^ ^255^ ^)^ Roowi (2010)^(^ ^256^ ^)^ Okello (2012)^(^ ^257^ ^)^ Ni (2008)^(^ ^258^ ^)^ Taguri (2006)^(^ ^259^ ^)^
	Pyrogallol	Antibacterial activity (especially against Gram-negative enterobacteria) An acetylcholinesterase inhibition greater than gallic acid parent Inhibition of *Vibrio* spp. quorum sensing	
	4-Hydroxyphenylacetic acid	Antimicrobial/antimycotic activity *in vitro*	
	(-)-5-(3′,4′-Dihydroxyphenyl)-γ-valerolactone	?	
Daidzein	Equol	Phyto-oeotrogen important for heart and bone health, and possible colon cancer protectants	Jackman (2007)^(^ ^260^ ^)^ Ishimi (2009)^(^ ^261^ ^)^ Davis (2009)^(^ ^262^ ^)^ Selma (2009)^(^ ^263^ ^)^
Daidzein	*O*-demethylangolensin	Oestrogenic and/or anti-oestrogenic activity	Larrosa (2006)^(^ ^264^ ^)^ Selma (2009)^(^ ^263^ ^)^
Quercetin	2-(3,4-Dihydroxyphenyl)acetic acid		Selma (2009)^(^ ^263^ ^)^
	2-3-(3-Hydroxyphenyl)acetic acid		
	3,4-Dihydroxybenzoic acid		
	Phloroglucinol		
	3-(3,4-Dihydroxyphenyl)propionic acid		
	3-(3-Hydroxyphenyl)propionic acid		
Kaempferol	2-(4-Hydroxyphenyl)acetic acid		Selma (2009)^(^ ^263^ ^)^
Naringenin	3-(4-Hydroxyphenyl)propionic acid	Antimicrobial activity against Gram-negative enterobacteria via outer membrane destabilisation	Selma (2009)^(^ ^263^ ^)^
	Phloroglucinol		
Isoxanthohumol	8-Prenylnaringenin	?	Selma (2009)^(^ ^263^ ^)^
Catechin and epicatechin										3-(3-Hydroxyphenyl)propionic acid		Alakomi (2007)^(^ ^254^ ^)^ Selma (2009)^(^ ^263^ ^)^
5-(3′,4′-Dihydroxyphenyl)-γ-valerolactone		
5-(3′-Hydroxyphenyl)-γ-valerolactone		
3-Hydroxyhippuric acid pyrogallol		
5-(3,4-Dihydroxyphenyl)valeric acid		
5-(3-Hydroxyohenyl)valeric acid		
3-(3,4-Dihydroxyphenyl)propionic acid	Antimicrobial activity against Gram-negative enterobacteria via outer membrane destabilisation	
5-(3-Methoxyohenyl)valeric acid		
3-(3,4-Dihydroxyphenyl)propionic acid		
5-(3-Methoxyohenyl)valeric acid		
2,3-Dihydroxyphenoxyl 3-(3′,4′-dihydroxyphenyl)propionic acid		
Ellagitannins/ellagic acid			Urolithin-A	Oestrogenic and/or anti-oestrogenic activity, antimalarials	Del Rio (2010)^(^ ^265^ ^)^ Larrosa (2006)^(^ ^264^ ^)^ Dell'Agli (2010)^(^ ^266^ ^)^
Urolithin-B	Oestrogenic and/or anti-oestrogenic activity, antimalarials	
Urolithin-C	Oestrogenic and/or anti-oestrogenic activity	
Urolithin-D	Oestrogenic and/or anti-oestrogenic activity	
Rutin	3-Hydroxyphenylacetic acid	Rutin and catabolites inhibit advanced glycation endproduct formation *in vitro* Antimicrobial activity against Gram-negative enterobacteria via outer membrane destabilisation	Alakomi (2007)^(^ ^254^ ^)^ Jaganath (2009)^(^ ^154^ ^)^ Cervantes-Laurean (2006)^(^ ^267^ ^)^
	3,4-Dihydroxybenzoic acid		
	4-Hydroxybenzoic acid		
	3-(3-Hydroxyphenyl)propionic acid		
	3,4-Dihydroxyphenylacetic acid		
Lignans	Enterolactone	Phyto-oestrogen important for heart and bone health, and possible colon cancer protectants	Davis (2009)^(^ ^262^ ^)^
	Enterodiol	Phyto-oestrogen important for heart and bone health, and possible colon cancer protectants	

Furthermore, the interaction between polyphenols and the microbiota is bi-directional. Recent evidence has shown that a number of polyphenols and their metabolites cause a selective stress or stimulus to some micro-organisms and influence other metabolic pathways like the production of SCFA^(^ ^152^ ^,^ ^153^ ^)^. In addition, *in vitro* incubation of faecal samples with quercetin-3-*O*-rutenoside (rutin) in the present of glucose as a carbon source showed a significant increase in deglycosylation of rutin and catabolism of quercetin, suggesting that prebiotic intervention might modify the bacterial metabolism of plant polyphenols^(^ ^154^ ^)^.

In metabolomic studies (see below) many of the compounds encountered relate to microbial catabolites of polyphenols that escape absorption in the small intestine^(^ ^155^ ^,^ ^156^ ^)^. These may become key markers of colonic bacterial activity.

### Emerging metabolites

A range of amino acids and related molecules, including tryptophan, γ-aminobutyric acid, γ-hydroxybutyric acid, or biogenic amines and also host–microbiota co-metabolic metabolites including catecholamines like dopamine and noradrenaline, and bile acids have the potential to make an impact both in a beneficial or harmful way with the host depending on concentration and chemical profile^(^ ^157^ ^)^. Recent scientific interest has been focused on microbiota production of cell-signalling molecules and neurotransmitters which through the gut–liver–brain axis appear to regulate a number of diverse physiological functions including energy intake and expenditure, brain development and cognitive function and mood^(^ ^158^ ^,^ ^159^ ^)^. However, by and large, human data are scarce and it needs to be evaluated whether prebiotic intervention might affect these signalling pathways.

## Factors that influence fermentation

The mechanisms controlling the metabolic activities of the colonic microbiota are only partly understood.

First of all, the type and quantity of dietary carbohydrate entering the colon have a dramatic impact on SCFA production. Many factors make an impact on the digestibility of carbohydrate in foods, not least their intrinsic chemical structure or biological availability in low-processed plant-derived foods. However, other food macromolecules ingested at the same meal, for example, red wine polyphenols or fat, and the load of complex carbohydrates like starch, can also determine the amount that reaches the colon^(^ ^160^ ^,^ ^161^ ^)^. Similarly, the extent of carbohydrate fermentation by the gut microbiota may be affected by other food components, for example, complex polyphenols, which may have antibacterial activity^(^ ^162^ ^)^.

Besides the diet, the specific phylogenetic and functional composition of the gut microbiota is influenced by a range of factors including host genetics, immunological factors and environmental factors including use of drugs such as antibiotics^(^ ^1^ ^,^ ^163^ ^)^. The large-intestinal microbiota has a strongly individual composition in human subjects and exhibits a remarkable compositional stability over time^(^ ^164^ ^)^, but is also amenable to dietary modulation^(^ ^165^ ^)^. Whereas some bacterial species are able to degrade a wide variety of substrates, other bacteria are nutritionally highly specialised^(^ ^1^ ^)^.

In addition, key geographic differences exist in bacteria and metabolites in different populations in different countries and regions, for example, in North and South China^(^ ^166^ ^)^, different countries in Europe^(^ ^163^ ^)^ and different populations (Afro-, Caucasian and Native-Americans) in the USA^(^ ^167^ ^)^. Some of these differences may be due to diet and lifestyle but genetic background may also be involved. These differences are important to take into account when investigating the relationship between bacterial metabolism and disease.

Finally, fermentation patterns are also determined by colonic transit times^(^ ^168^ ^)^. In a study by Cummings *et al.* a significant correlation was observed between colonic transit times and urinary excretion rates of phenols^(^ ^169^ ^)^. With shorter transit times, turnover rate is faster and microbial growth is more efficient, resulting in a greater mass of bacteria^(^ ^170^ ^)^. Similarly, in *in vitro* fermentation experiments with faecal inocula from volunteers with pharmacologically modified transit times, reduction of transit time was associated with increased production of SCFA and increased disappearance of substrate^(^ ^171^ ^)^. Although fermentation of carbohydrate occurs mainly in the proximal colon, a mixture of fermentable and less fermentable carbohydrates in the diet can push fermentation further around the colon and thus increase SCFA also in the more distal parts of the colon^(^ ^172^ ^–^ ^175^ ^)^. The European Food Safety Authority has recently approved two health claims for wheat bran in relation to two beneficial physiological effects, namely an increase in faecal bulk and a reduction of intestinal transit time. Whether increased SCFA production is responsible for the increased transit time in humans remains to be studied. In animal models, SCFA inhibit peristaltic contractile activity, however, only at concentrations above a physiological threshold^(^ ^176^ ^)^. In addition, the motor effects of SCFA may differ between species as intracolonic infusion of a 100 mm-SCFA solution did not modify transit in two healthy human subjects^(^ ^177^ ^)^. In a recent study in ten volunteers, infusion of a 100 mm-SCFA solution did not affect the phasic or tonic motor activity of the colon or the number of high-amplitude-propagated contractions^(^ ^178^ ^)^.

## Gaps and limitations

### Where to measure: choice of the biomatrix

A major obstacle in the evaluation of intestinal bacterial metabolism *in vivo* in human subjects is the inability to directly sample at the site of production. Therefore, much information has been obtained from analysis of faecal samples and supportive data from *in vitro* and experimental models. However, information on the activity of the intestinal microbiota can be derived from analysis of various biological samples including faecal samples, serum or plasma and urine samples. Zhao *et al.* ^(^ ^174^ ^)^ nicely showed that a modification of the intestinal microbiota in mice resulted in altered metabolite patterns in faeces. Administration of non-absorbable antibiotics resulted in increased levels of *Bacteroides* and *Enterococcus* species and was accompanied by a reduction in the overall fermentation of indigestible carbohydrates with lower levels of SCFA, lower levels of many amino acids and a disturbance of bile acid metabolism^(^ ^174^ ^)^. In contrast, the faecal metabolome in horses was shown not to be representative of the colonic metabolome^(^ ^179^ ^)^, which reflects the different patterns of bacterial metabolism and the absorption of products in different parts of the colon. Similarly, the impact of microbiota activity is reflected in the levels of several serum metabolites^(^ ^180^ ^)^. Interestingly, the host responds to many of those metabolites with phase II metabolism comparable with the response to drugs, as many metabolites are sulfated, glycine conjugated or glucuronidated to facilitate urinary excretion.

In recent years, many research efforts have focused on the mechanisms by which the SCFA acetate, propionate and butyrate affect host physiology, Nevertheless, reliable and quantifiable methodologies have rarely been employed to measure the relative SCFA production for different fibres in human subjects, or to quantify their relative contribution to circulating SCFA pools, for example, using stable isotope tracking or pharmaco- or nutri-kinetic approaches^(^ ^181^ ^)^. In addition, very few studies have examined the time course of SCFA production, absorption and utilisation after prebiotic intervention or examined the impact of other food components, underlying host disease or gut microbiota composition and genetic potential on these processes. Therefore it has been difficult to convincingly prove in human subjects a role for colonic SCFA produced from prebiotics in the key physiological processes proven to be regulated by SCFA in animal models. There is a critical need for multidisciplinary studies to address these questions and take that final mechanistic step from animal model to human physiology and health.

### When to measure: snapshot analysis

Intestinal microbial fermentation is a dynamic process influenced by a wide range of factors (see above). Therefore, the nature and concentration of metabolites produced by the microbiota is context-dependent and the levels of each metabolite are a result of metabolic fluxes of highly variable rates, which are not adequately represented in steady-state metabolite profiles. The rapid uptake and conversion of metabolic intermediates, as well as their removal from the intestinal lumen through host absorption creates a highly dynamic system, which is strongly discrepant with the methodologies for analyses that can only provide a single time point quantification or ‘snapshot’. At present, very little is known about the short- or long-term variation in metabolite concentrations produced by the gut bacteria. The dynamic analysis of metabolic conversions within the microbiota may be further unravelled through the application of stable isotope-labelled nutrients^(^ ^182^ ^)^ that, in combination with metabolic modelling (for example, including the use of meta-transcriptome or meta-proteome datasets), may enable the determination of metabolic fluxes in the microbiota and the host mucosa.

## More holistic approach

### Functional analysis of faecal water

Prebiotics can affect the levels of many compounds in the gastrointestinal tract. These may be constitutively produced in the body, such as bile acids, or may be bacterial metabolites, such as SCFA. This complicates assessment of the potential effects of prebiotics on human health based on evaluation of individual substances. Hence, to supplement such assessment, a more holistic approach to the effects of prebiotics on the biological activity of the gastrointestinal milieu is required. One approach that might be useful for this purpose is the functional analysis of faecal water (for a review, see Osswald *et al.* ^(^ ^183^ ^)^). This should provide an integrated measure of the overall contribution of the compounds present to a defined biological endpoint, such as genotoxicity.

Faecal water has a number of potential advantages in such studies. It is non-invasive, it can reflect the effects of diet directly, it samples the initial target compartment, the gastrointestinal tract, it provides a measure of the total activity of what was present in the distal colon, it can provide repeated measurements over time, and subjects can serve as their own controls. Disadvantages include the practicalities of sample collection and the reluctance of some subjects to provide samples, the complexity of the biofluid may interfere in the assessment of some endpoints and it may not always be truly representative of the biological compartment of interest, because of modulation of intestinal contents before faecal excretion.

Perhaps the endpoint most widely assessed using faecal water is genotoxicity. A number of assays have been used for this purpose, including the Ames *Salmonella* test and SOS Chromo test for bacterial mutagenicity^(^ ^184^ ^–^ ^187^ ^)^. Over the past 15 years, those bacterial mutagenicity assays have been almost completely replaced by assays using mammalian cells as targets^(^ ^187^ ^)^. The Comet assay for DNA strand breaks in enterocyte cells (Caco-2, HT-29 and Hep G2 (liver derived)) has been commonly used to assess the genotoxic potential of faecal water. The considerable inter- and intra-individual variability between samples and individuals, possibly reflecting the effects of dietary variation, constitutes a major limitation. Use of a controlled dietary regimen for 9 d was of insufficient length to reduce interindividual variability, suggesting that changing the genotoxic potential of the gut microbiota may require much longer^(^ ^188^ ^)^. There is no ready means for normalisation of sample activity, equivalent, for example, to creatinine in urine. Efforts to use wet or dry weight of the stool have met with little success. However, the method of preparation of faecal water has little effect on the biological measurement that facilitates comparison across studies^(^ ^103^ ^)^.

The toxicity of faecal water has been assessed using a number of endpoints. Cytotoxicity has been measured by colorimetric cell viability assays based on the cleavage of a tetrazolium salt (for example, 3-(4,5-dimethylthiazol-2-yl)-2,5-diphenyltetrazolium bromide (MTT) or water-soluble tetrazolium salt 1 (WST-1)) in the mitochondria of living cells to a coloured formazan derivative^(^ ^189^ ^,^ ^190^ ^)^. Changes in barrier function are assessed from impairment of tight junctions using transepithelial resistance^(^ ^191^ ^,^ ^192^ ^)^ or phenol red leakage^(^ ^193^ ^,^ ^194^ ^)^ in Caco-2 cells, and invasive potential of HTC116 cells using fluorescence-activated cell sorting (FACS) analysis^(^ ^191^ ^)^. In addition, the lytic activity of faecal water to erythrocytes has been quantified as a parameter of cytotoxicity and was significantly correlated to colonic cell proliferation^(^ ^195^ ^)^. Finally, the effects of faecal water on apoptosis rates in the human colon-derived cell lines HT-29 and FHC (fetal human cells) have been evaluated^(^ ^196^ ^)^. A number of apoptotic hallmarks were measured: changes in cell morphology, DNA fragmentation, FACS analysis of DNA strand breaks assessed using the terminal deoxynucleotidyl transferase dUTP nick end labelling (TUNEL) assay, and poly(ADP-ribose) polymerase cleavage.

In addition, several assays have been used to measure the effects of faecal water on cell proliferation. Using two human colon carcinoma cell lines, HT-29 and HCT 116, faecal water or their lipid extracts were found to activate activator protein-1 (AP-1), a transcription factor associated with the promotion of neoplastic transformation^(^ ^197^ ^)^, and to induce COX-2 promotor activity, which has been implicated in colon carcinogenesis^(^ ^198^ ^)^. Faecal water also inhibited cell cycle progression in HT-29 cells and down-regulated the gene expression of proliferating cell nuclear antigen, a protein essential for replication^(^ ^199^ ^)^.

Results on the effects of oligofructose and/or inulin treatment on the genotoxicity of faecal water in rats have been equivocal^(^ ^200^ ^)^. In a study in rats treated with azoxymethane, DNA damage assessed using the Comet assay was reduced with faecal samples obtained after 4 or more months of prebiotic treatment. Evidence from this study suggested that the antigenotoxic effects of prebiotics occur rapidly and that azoxymethane-induced tumour development increases the genotoxicity of faecal water^(^ ^201^ ^)^.

Relatively few studies to date have evaluated the effects of prebiotic administration on the biological effects of faecal water samples from human subjects. Feeding male smokers and non-smokers either plain sourdough bread, bread supplemented with prebiotics (inulin, linseed and soya flours) or bread additionally supplemented with antioxidants resulted, in the non-smoker group, in about 50 % reduction in DNA strand breaks induced by faecal water with both the control and test breads^(^ ^202^ ^)^. In smokers, the control and test breads reduced faecal water genotoxicity only in those with the glutathione *S*-transferase genotype GSTM1*0. Administration of polydextrose (8 g/d for 3 weeks) reduced faecal water genotoxicity using the Comet assay in thirty-three healthy subjects in a double-blind placebo controlled cross-over study^(^ ^44^ ^)^. Similarly, supplementation of the diet with konjac glucomannan (4·5 g/d) for 4 weeks in thirty healthy subjects significantly reduced faecal water genotoxicity^(^ ^203^ ^)^. In contrast, no changes in faecal water genotoxicity were observed after 4 weeks treatment with galacto-oligosaccharides (4 g/d) in a population above 50 years^(^ ^204^ ^)^.

Measurement of biological activity of faecal water samples is an attractive means of linking changes in the colonic contents with health outcomes. However, there are a number of potential limitations to this methodology. Standardisation of the assay protocols in terms of target cells and sample preparation is mandated to allow comparison of data between studies. To date there have been few studies using this approach in the evaluation of prebiotics.

### Metabolomics

Metabolomics offers an alternative and holistic approach to understanding the interaction between the human gut microbiome and host metabolism as well as to identify possible biomarkers of gut health. Metabolomic studies allow simultaneous evaluation of a wide range of metabolites by a top-down approach bypassing the need for an *a priori* hypothesis. Several analytical platforms allow detection, identification and quantification of different ranges of molecules, including ^1^H-NMR, LC-MSMS and GC-MS^(^ ^205^ ^,^ ^206^ ^)^. However, due to the chemical diversity and different physico-chemical properties of the metabolites and the large dynamic range of metabolite concentrations in different biological samples, it is virtually impossible to measure the complete metabolome. In addition, the term ‘metabolome’ can refer to faeces^(^ ^205^ ^)^, urine or plasma^(^ ^207^ ^)^. By selecting a specific analytical platform and a biofluid in which metabolites will be measured, the metabolome will be reduced to those specific conditions. Metabolome approaches can be non-targeted so that any compounds are considered in a pattern, or targeted where more specific types of molecules are identified and sometimes quantified. In general, however, the strategy is to discover patterns of metabolites which are associated with disease states such as cancer^(^ ^208^ ^–^ ^210^ ^)^, the metabolic syndrome^(^ ^211^ ^)^, obesity^(^ ^212^ ^)^, CVD, diabetes^(^ ^213^ ^)^, gut disease such as UC^(^ ^205^ ^)^, irritable bowel syndrome^(^ ^214^ ^)^, peptic ulcer^(^ ^215^ ^)^ and the impact of changes in body weight or diet^(^ ^156^ ^,^ ^211^ ^,^ ^216^ ^,^ ^217^ ^)^.

Although broad-spectrum metabolomics is shedding new light on the metabolites derived from the gut microbiota at an unprecedented resolution, compound quantification has been found time-consuming and has not been a priority in many metabolomics protocols. However, there is still a place for accurate, reproducible and targeted analytical chemistry approaches to quantify selected panels of health-relevant metabolites present in various body biofluids including faeces. Accurate quantification is particularly important when determining subtle changes in metabolite levels in response to dietary interventions which rarely block or ‘turn-off’ pathways or metabolic activities as drugs do but rather modulate the production rate of metabolites. Advances in mass spectroscopy instrumentation and methodology have allowed the development of ‘targeted metabolomics’ approaches where there are panels of specific metabolites, often of related physiological relevance, as an example metabolites related to diabetes or dyslipidaemia, can be accurately and reproducibly quantified in blood and urine^(^ ^218^ ^)^.

Application of metabolomic analysis in the evaluation of putative health benefits of prebiotics might provide additional evidence or elucidate the molecular bases of their actions. Martin *et al.* used a model of mice colonised with a human baby's microbiota to evaluate the impact of prebiotic galactosyl-oligosaccharides on the metabolic changes in ten biofluids/compartments. Prebiotic administration significantly reduced the lipids in the liver and kidneys and altered the transmethylation metabolic pathways (homocysteine–betaine)^(^ ^219^ ^)^. So far, only a few intervention studies in human subjects applied metabolomics to evaluate the impact of a synbiotic^(^ ^220^ ^–^ ^222^ ^)^. Recently, a metabolomic approach was applied in a placebo-controlled trial in patients with Crohn's disease who received a dietary intervention with oligofructose-enriched inulin (OF-IN) (2 × 10 g/d) for 4 weeks^(^ ^223^ ^)^. Faecal butyrate levels were up-regulated to the levels found in faecal samples of healthy controls after OF-IN intake. In view of the immunomodulatory and anti-inflammatory properties of butyrate, these observations might encourage follow-up studies in Crohn's disease with prebiotics.

### Link to metagenome analysis

Meta-omics technologies enable the simultaneous, ecosystem-wide readout of phylogenetic composition and function, providing a blueprint of the microbiota's functional potential. Metagenomics employs established technologies that are fuelled by the continuous advances made in sequencing technologies, allowing the cost- and time-effective creation of functional catalogues of the human intestinal microbiota in individuals^(^ ^2^ ^)^, which have confirmed a substantially distinct microbial community composition in the large *v.* the small intestine^(^ ^224^ ^)^. It is far from trivial to translate the genetic repertoire or the metagenome of the microbiota into its actual *in situ* activity, and there is a strong requirement for the further development of functional metagenomic approaches, including metatranscriptomics and metaproteomics. Finally, meta-metabolomics/metabonomics approaches (on faecal water, blood or urine) allow monitoring the pool of metabolites produced by the microbiota in the intestinal lumen (faecal water metabolomes), and their impact on the host's systemic biochemistry (urine and blood metabolomes).

A main bottleneck in these approaches is the biological interpretation of these data and their integration to decipher the underlying interactions. All the techniques mentioned above yield massive datasets with large proportions of unknown features (unannotated genes, unidentified spectra, etc.). In addition, all data are fragmented and include the assignment of incomplete spectra to fragmented gene sequences and partially sequenced species, which is a challenging problem even for meta-omics datasets that are generated from the same samples. Also the integration of metabolomic profiles and intestinal metagenome composition remains challenging, which is also a consequence of the ‘snapshot characteristics’ of the datasets generated.

**Fig. 2:**
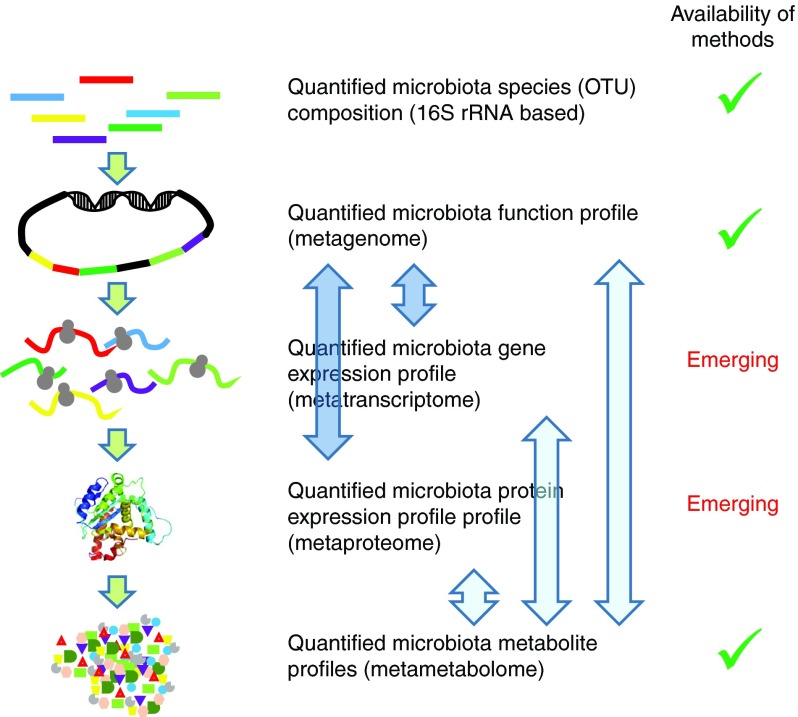
Schematic presentation of the future needs for the functional analysis of the microbiota. Metagenome mapping of metatranscriptome and metaproteome data can rely on established methodologies (darker arrows), but the integration to these (functional) metagenome data with the meta-metabolome is far from trivial and in need of methodology development (lighter arrows). OTU, operational taxonomic units. A colour version of this figure can be found online at http://www.journals.cambridge.org/nrr

Fig. 2 shows a schematic presentation of the future needs to analyse the functional capacities of the microbiota.

Bioinformatic processing and interpretation of large-scale metabolic datasets are hampered by the inherent broad scope of the current metabolic databases (for example, KEGG (Kyoto Encyclopedia of Genes and Genomes), etc.). These generic metabolic maps contain pathways that are not present in the gut ecosystem (for example, reactions requiring molecular oxygen as a substrate) and at the same time lack gut-specific pathways, which leads to a loss of both sensitivity and specificity in meta-omics data interpretation. This situation implies that there is a great need for gut-specific, specialised and curated databases, combined with dedicated software and visualisation tools to facilitate the effective interpretation of meta-omics datasets. These bioinformatic environments are under construction (J Raes, personal communication) and will be of great value for the metabolic deciphering of microbiota adaptations to dietary or pre-/pro-/symbiotic interventions. The detailed inference of metabolic potential and activity, when combined with species-function mapping and reconstruction of metabolic interactions between microbial groups, including the reconstruction of syntrophic chains and metabolite exchange^(^ ^225^ ^)^, will open the way towards novel strategies in mathematical modelling of the metabolic processes taking place in the intestine.

The challenges of data integration become even more pronounced when the intestine meta-omics data are to be connected to parameters that relate to the host's physiology. Some of these host analyses can encompass high-resolution measurements like blood or urine metabolite profiles, blood transcriptomes, or peripheral blood analyte patterns (cytokines, chemokines, hormones, etc.). Connecting these host measurements to meta-omics data is far from trivial, for example, because for many metabolites detected in blood or urine it is uncertain whether they are of intestinal origin. As a consequence, current studies usually limit themselves to the descriptive analysis of metabolic potential and/or activity across patient cohorts, in which the integration is commonly limited to the detection of correlated entities within the datasets that can be identified by multivariate statistics. Only in a few cases were the identified correlations explained through biological context and network biology reconstructions that explain the molecular relationships between the observed correlations. The latter process commonly requires a time-consuming and largely manual sifting of results combined with massive literature mining to decipher the biological context of the observed correlations. Systems biology mathematical frameworks that accelerate the conversion of correlation-based mining to comprehensive, hypothesis-generating biological interpretation, could accelerate the progress of the meta-omics field and its relevance in human health and disease. However, these computational frameworks are still in their infancy, and will require a substantial amount of validation before they can reliably be applied to effectively mine the complex multivariate datasets obtained through meta-omics and high-resolution host analyses.

## Conclusions

Currently, there is insufficient evidence to use changes in levels of individual bacterial metabolites as markers in the assessment of prebiotic effectivity. Several *in vitro* and experimental animal studies indicate that protein fermentation metabolites including ammonia, phenol, *p*-cresol, indole or hydrogen sulfide intrinsically affect epithelial cellular metabolism and barrier function. However, there is no evidence from human studies that a reduction in faecal excretion of those metabolites contributes to health. Possibly, the impact of protein fermentation is overshadowed by other dietary or lifestyle factors. Although SCFA are generally recognised as markers of carbohydrate rather than protein fermentation in the colon and are therefore commonly considered as beneficial to health, a number of critical questions need to be answered before their concentrations can serve as biomarkers.

In particular, the lack of reliable concentration ranges defining the ‘normal’ or healthy state for these different metabolites in faeces and other biofluids, and the fact that steady-state metabolite concentrations or profiles do not take into account the rapid absorption and/or conversion of the metabolites, hampers the routine application of those techniques to human dietary interventions where microbiota modulation is an objective. There is an urgent need for dynamic, nutrikinetic-type studies, for example, with stable isotopes, to determine and quantify the pathway of microbial metabolites into the different body compartments. Functional analysis of faecal water toxicity has been proposed as a more holistic approach to link changes in colonic content to health outcomes but suffers from some practical considerations and the limited validation of this biomarker towards the end point of colorectal cancer.

Despite the challenges encountered in the integration of the different levels of quantitative analyses of the intestinal system through meta-omics and the corresponding host-specific parameters, the available meta-omics and other high-resolution analytical methods enable the determination of correlated multivariate signatures that can place potential metabolic or health markers in their context, thereby enhancing their value as markers in health and disease or in therapy efficacy evaluation. Of course, these meta-omics must first consider the prevailing ‘meta-data’ which govern nutrient concentrations within human biofluids, not least dietary intake, a difficult parameter to measure and control in free-living subjects. However, these developments may significantly refine our views of concepts like ‘the bandwidth of health’^(^ ^226^ ^)^ that postulate that multiple molecular solutions for a healthy functioning mucosa and/or microbiota exist. The multivariate signatures mentioned may enable appropriate population stratification for the more effective application of specific nutritional interventions in subpopulations that are predictably more responsive to a certain treatment. Meta-omic stratification of the human population is illustrated by the distinction of three ‘metagenomic enterotypes’ that are characterised by elevated community sizes of the Bacteroidetes, *Prevotella* and ruminococci^(^ ^227^ ^)^. Taken together, the deciphering of detailed and specific mechanisms of interaction in the host–microbe-metabolic interplay are a challenge for the future, but hold great promise for rationalised nutritional health improvement and/or even disease therapy in stratified population cohorts.
